# Simple and Complicated Regulating Mechanisms; Æsthetic Mechanisms; Proper and Improper Anchorage

**Published:** 1895-07

**Authors:** J. N. Farrar

**Affiliations:** New York City.


ARTICLE IV.

### SIMPLE AND COMPLICATED REGULATING MECHANISMS; ÆSTHETIC MECHANISMS; PROPER AND IMPROPER ANCHORAGE.

BY J. N. FARRAR. M. D., D. D. S., NEW YORK CITY.

*Simple and Complicated Regulating Mechanisms.*—In some of my published papers I have dwelt somewhat on this subject; but as there are some *dentists* who persist in efforts to *prejudice students against improvements* in regulating mechanisms, a few more remarks will be made. In mechanics simplicity is the great desideratum with all natural inventors. No useless elements are found in first-class machines of any kind; but it is not everybody who understands this subject sufficiently to comprehend that there can be simplicity in complicated machinery, and that some of the grandest and most valuable machines ever invented are so because of this complicated simplicity. The Hoe printing-press is a startling example of this. More than 16,000 pieces in one machine, printing, folding, cutting, pasting and filing 96,000 newspapers per hour, is an example of complicated simplicity and a marvelous illustration of the fruits of the genius of man. The true mechanical genius *laughs to scorn* those who attempt to ridicule the best mechanisms ever invented for correction of irregularities of the teeth, calling them unnecessarily complicated, simply because they themselves have not sufficient skill to even copy them. So long as the dental profession contented itself with crude, cumbersome mechanisms, no progress was made in this branch; but when higher mechanical skill was applied it advanced and continued to advance as more and more thought was centered upon the subject, until now it has loomed up as one of the greatest of all the branches of dentistry. Indeed, it has reached a standard that never could have been attained from the acceptance of the views of those persons who are always to be found pulling back until dragged forward by public sentiment. In my office there are hundreds of mechanisms, many of which may at first seem to the tyro to be more complicated than is necessary; but there is not one that is really complex to an expert. Those which at first appear so are only examples of complicated simplicity that operate as easily as the turning of a screw. There are in vogue old-fashioned regulating mechanisms, consisting of plates and springs, match-sticks and strings, that *are* complex. These are not only difficult to manage, taking unnecessary time and causing great pain to change the strings, but impossible to be kept clean. With a lot of such things matted with decomposed food, no patient can feel happy over an operation.

True mechanics recognize any mechanism as simple that can be operated easily and will accomplish the result desired. Anything that requires great care and unnecessary time to manage is regarded by them as an imperfect machine. In other words, a regulating mechanism, if difficult to manage and painful to endure, must be regarded as more complicated than one (though requiring manipulative, mechanical genius to contrive) that will, when properly finished and applied, be managed easily and quickly, without causing pain. The former is *low mechanics*, the latter *high mechanics*.

*Æsthetic Mechanism.*—Another phase of high mechanics, as applied to dentistry, is the æsthetic. Failure may arise from lack of skill on the part of the operator or by perversity of the patient. But many failures can be traced solely to the disgust of patients through lack of æsthetics in the mechanisms, because conspicuously black and filthy mechanisms do not encourage the patients. There have been many failures, by the use of such things, that would have ended in a glorious success if delicately and beautifully made gold mechanisms had been used.

*Anchorage, Proper and Improper.*—When the first molar has been extracted it becomes necessary to use the second and third molars for anchorage, to move back the bicuspids; but they should not be drawn upon for moving more than one tooth at a time, and even then these anchor teeth should be frequently examined to prevent too great inclination forward. If they have moved too far, they should be given liberty, permitting them to return to their normal positions. It should not be inferred from this, however, that the starting forward only a short distance is dangerous to these teeth, as all anchor teeth are liable, and even expected, to be moved somewhat by the draught. The question to decide is, how long such started anchor teeth can be safely used to further the operation. If the antagonism is not broken too much, these teeth will generally settle back into their former and normal places, even if they are elevated slightly in their sockets. If elevated, however, it may require months. Because of this natural tendency to settle back and down into the sockets, it is *not prudent* to be in too great haste to *grind teeth* to improve antagonism after having completed the operation of moving teeth.

In planning a mechanism for moving teeth, one of the main points to be taken into consideration is enduring, firm anchorage. In my own practice I always include for anchorage all the teeth that are practicable, not only to prevent their being moved, but also to prevent soreness of the sockets. The exceptions to this rule of practice are in cases where a sufficient number of teeth cannot be taken advantage of for anchorage, or where it is necessary to *move anchor teeth forward to aid in filling extra space.—Ohio Dental Journal.*

